# Between Order and Confusion: Clearing up Structural Misconceptions in Carbon Materials Nomenclature

**DOI:** 10.1002/anie.202519975

**Published:** 2026-02-03

**Authors:** Chantal Glatthaar, Felix Badaczewski, Peter J. Klar, Bernd M. Smarsly

**Affiliations:** ^1^ Institute of Physical Chemistry Center of Materials Research Justus Liebig University Giessen Giessen 35392 Germany; ^2^ Schunk Kohlenstofftechnik GmbH Heuchelheim 35452 Germany; ^3^ Institute of Experimental Physics I Center of Materials Research Justus Liebig University Giessen Giessen 35392 Germany

**Keywords:** Carbon, Nomenclature, Raman spectroscopy, X‐ray scattering

## Abstract

Carbon materials are of increasing importance for various applications especially in the field of energy storage and conversion as well as electrocatalysis, and thus, they are essential for the worldwide transition toward an emission‐free economy. Hence, various carbonaceous materials with different levels of order of graphene stacks are discussed in current literature. However, precise characterization or even the correct terminology of the respective material is often neglected, leading to miscommunication and misinterpretation throughout the carbon community, especially with respect to “amorphous” carbon. Here, we aim to clarify the terms describing different graphene‐containing carbon materials, namely graphite, graphitic carbon, non‐graphitic carbon, and amorphous carbon. We recall the IUPAC definitions of these materials and correlate them with characteristic X‐ray scattering patterns as well as Raman spectra, being the most widespread and appropriate characterization techniques. Thereby, this scientific perspective strives toward raising awareness for fine structural differences and the need for a consistent characterization and description of these carbon materials.

## Graphical abstract

Despite the versatile use of carbon materials, inconsistent terminology and inadequate characterization – especially regarding graphene structures based on sp^2^‐hybridized carbon atoms – often cause confusion. This perspective clarifies terms like graphite, graphitic carbon, non‐graphitic carbon, and amorphous carbon by aligning IUPAC definitions with X‐ray scattering and Raman data, highlighting how structural differences impact material characterization and classification.

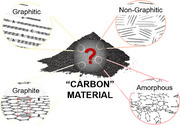

## 1 Introduction

**Figure 1:**
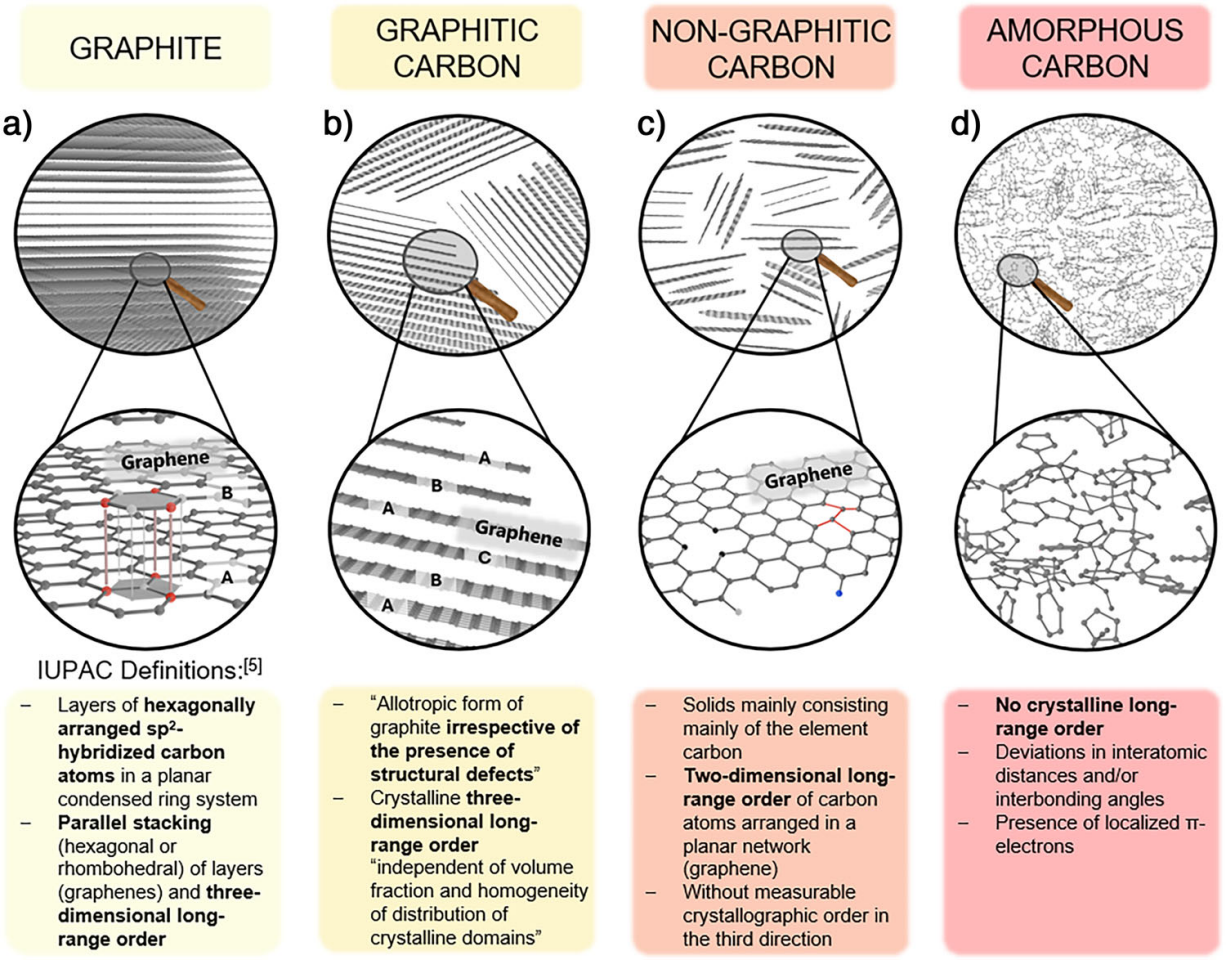
Schematic illustration of the atomic arrangement within the discussed carbonaceous materials together with the IUPAC definitions^[^ ^5^ ^]^ below. In graphite a), graphene layers are stacked in an ABAB hexagonal way as shown here. Alternatively, rhombohedral stacking is possible as well. In graphitic carbon b), the graphene layer stacking is not as regular as in graphite as indicated with the exemplary ABCABA stacking. For non‐graphitic carbon c), no 3D long‐range order is present and within the graphene layer defects like sp^3^‐hybridiced carbon atoms (black), hydroxy (light gray) or amine (blue) groups, or varying C─C bond lengths (red) can occur. Amorphous carbon d) is illustrated with random sp^2^ and sp^3^‐type carbon assemblies.

The element carbon governs not only the field of organic chemistry but is also a key element for materials science, in general. Carbon materials based on stacks of roughly planar polyaromatic entities (“graphene‐like” layers, see Figure 1) are highly relevant and used on a million‐ton scale. Such so‐called “graphitic” and “non‐graphitic” carbon materials, based on nanoscaled graphene layers as the basic structural unit, stand out due to their properties such as an adjustable nanoscale structure, high conductivity, inertness, but also due to abundance and low price.^[^ ^1^ ^]^ Their characteristics enable versatile applications in, *i.e*., separation, energy conversion in fuel cells or energy storage in batteries as well as supercapacitors.^[^ ^1^, ^2^, ^3^, ^4^ ^]^ For each application, researchers have developed various distinct types of such graphene‐based carbonaceous materials differing in their carbonization treatment, porosity, or nanoscaled structure. Concomitantly, several terms describing the respective type of carbonaceous materials entered the literature. “Glass‐like carbon”, “hard carbon”, “soft carbon”, as well as “activated carbon” are just some prominent expressions that have been established based on a typical macroscopic property (“hard” carbon) or a particular preparation procedure (“activated” carbon). These terms are applied to classify a subgroup within the rich and still expanding class of carbonaceous materials. Certainly, a categorization in this dynamic field of material research is useful and even imperative. For instance, by dubbing a material as “activated carbon” it is generally agreed on that this carbon material has undergone an “activation” process and features a high surface area. However, in regard to the structure and order on the atomic scale, the existing precise classification should be used. It is the well‐defined and coherent IUPAC terminology for carbon materials based on the graphene unit as building block, which is founded on apparent fundamental structural characteristics on the atomic or nanoscale. Unfortunately, often IUPAC terminology is either ignored or applied incorrectly in numerous publications. Partly, a confusion in literature might originate from the semantic of official IUPAC terminology for the different carbon‐based materials. Although describing materials possessing significantly different degrees of structural order, the terms “non‐graphitic”, “graphitic”, and “graphite” have a high phonological similarity possibly promoting confusion. Moreover, all these materials are built of graphene layers, whereas “graphene” describes the single carbon layer as basic structural unit, but at the same time also a class of a unique 2D material – discovered in 2004 – consisting of only one single layer of hexagonally arranged and planar condensed ring system of carbon atoms, exclusively (see Figure S1).^[^ ^5^, ^6^ ^]^ Anyhow, the ignorance or incorrect application of the IUPAC terminology may lead to an incorrect allocation and, consequently, to even false interpretation in terms of structure‐property relationship. Here, drawing attention to the inconsistency in nomenclature is more than pointing at terminological formalities. In fact, ignorance or misutilization of the IUPAC nomenclature leads to misconceptions as well as improper conclusions about the relationship between the structure of carbon materials on the atomic scale and properties. Especially when it comes to the relationship between electrochemistry and structural features, the misutilization causes various problems:

First, applying non‐official terminology and nomenclature, a certain class of carbon materials might be called differently in scientific publications. For instance, “hard carbon” is a widespread term, but is not included in IUPAC terminology, which itself does not necessarily result in confusion. Yet, on the one hand, “hard carbon” is identical to a “non‐graphitizable carbon” (which is the correct IUPAC nomenclature), the latter being more suitable for a precise classification. On the other hand, unfortunately, “hard carbon” is often associated with an “amorphous” structure on the atomic scale,^[^ ^7^, ^8^ ^]^ which is incorrect and results in misconceptions. Also, “hard carbon” should not be denoted as “graphitic” carbon in order to describe the general existence of graphene‐like layers and stacks as basic structural motif.^[^ ^9^ ^]^ Accordingly, also Monthioux^[^ ^10^ ^]^ criticized the usage of the term “graphitic” in that sense most recently. This is not only a formal misuse since the term “graphitic” – by IUPAC definition – describes a high degree of order very close to graphite which is not the case for “hard carbon”. Such a wrong designation of carbonaceous materials which is not in accordance with IUPAC can result in the association with an incorrect picture of the microscopic structure. As soon as an incorrect structural assembly of graphene layers is assumed, wrong conclusions from scientific experiments can be drawn. Consequently, the answer to the question of the best suited structure for specific applications of the utilized carbonaceous material might be unattainable. Thus, an entire field of research may steer in the wrong direction, miscommunication is transferred across publications, and scientific progress is hampered.

Second, again taking “hard carbon” as example, the macroscopic property “hardness” is used to infer a distinct microscopic structure. However, this macroscopic‐microscopic correlation is not unambigous, *i.e*., several materials with different microscopic configurations can exhibit similar macroscopic hardness. Thus, structural classifications should not be based on macroscopic observation in order to avoid incorrect structural assumptions.^[^ ^10^ ^]^

To clarify differences in atomic structures, we discuss the structure of graphite, graphitic carbon, non‐graphitic carbon, and amorphous carbon on the atomic scale, according to IUPAC classification, which is founded on the differences in the order of the polyaromatic hexagonal layers of carbon atoms and their stacking. Furthermore, we correlate the structural properties with the signatures found in diffraction/scattering patterns of X‐rays or neutrons and Raman spectra. We focused on these two techniques as they present the most widespread structure‐elucidating methods providing meaningful average structural parameters on the nanometer scale by standardized evaluation procedures, being representative for the bulk material, in contrast to, *i.e*., the local method of high resolution transmission electron microscopy (HRTEM).^[^ ^10^ ^]^ With our overview of how to characterize different carbonaceous materials, we intend to help carbon researchers to better understand their materials and to avoid structural misjudgments. Stressing a clear terminology, we want to contribute to an improvement of communication between scientists which is the basis for advancing carbon research.

## 2 From Graphite to Amorphous Carbon–Classification of Different Carbon‐Based Materials

### 2.1 Graphite

**Figure 2:**
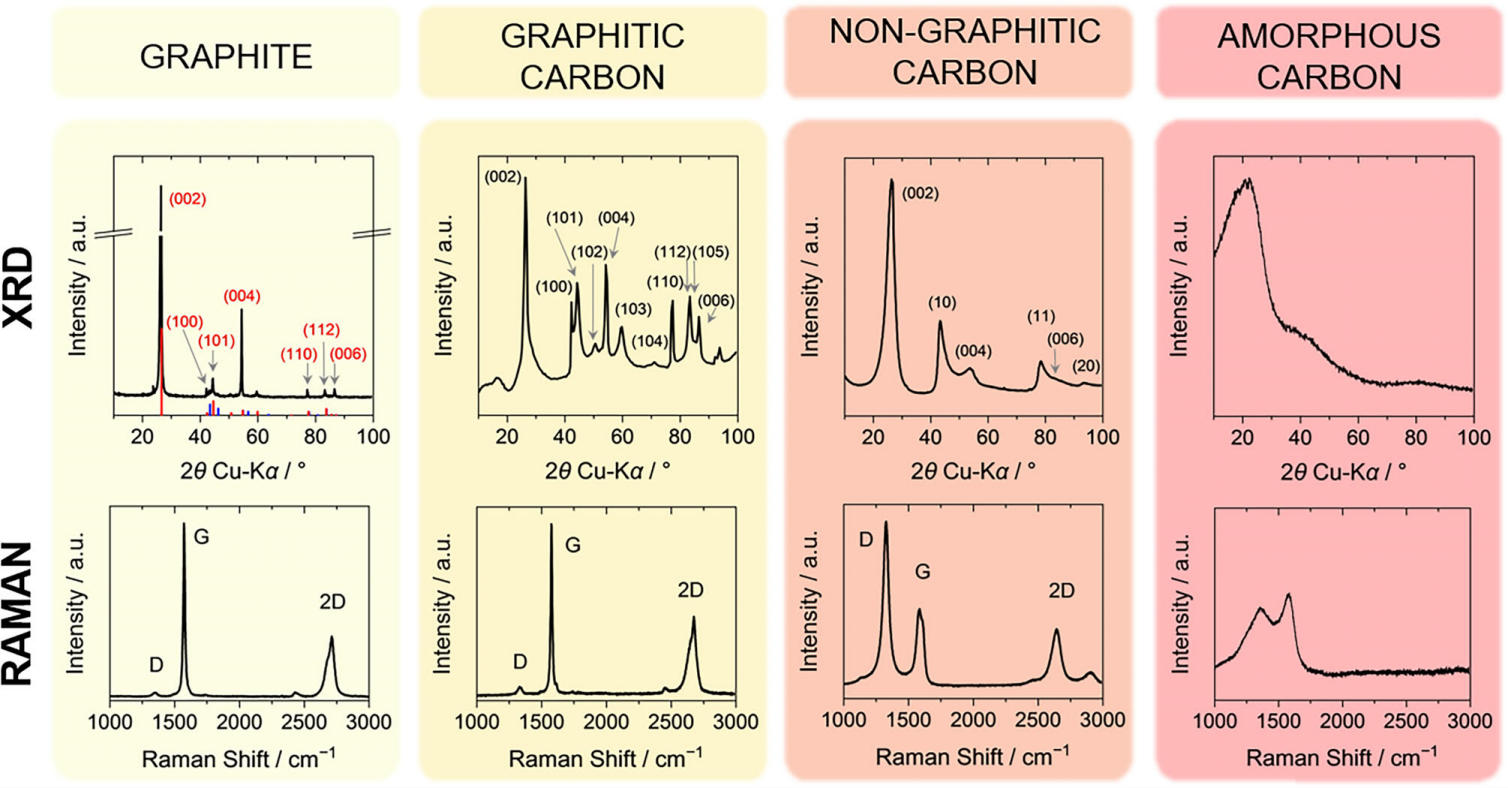
Characteristic X‐ray scattering patterns (top row) and Raman spectra (bottom row) of representative samples of each type of material. Graphite patterns with hexagonal (red, ICSD 98–003–1170) as well as rhombohedral (blue, ICSD 98–003–1829) graphite references are given (left). A low softening‐point pitch processed at 3000 °C serves as example for graphitic carbon (middle left), the X‐ray scattering pattern of which was reprinted with permission from Ref. [26] (Copyright 2016, Elsevier), and Raman spectrum was reprinted with permission under a Creative Commons CC‐BY 4.0 from Ref. [11] (Copyright 2020, Elsevier). As representative for a non‐graphitic carbon (middle right), a phenol‐formaldehyde resin treated at 3000 °C was chosen. The X‐ray scattering pattern was reproduced with permission from Ref. [27] (Copyright 2019, Elsevier), and Raman spectrum was reprinted with permission under a Creative Commons CC‐BY 4.0 from Ref. [11] (Copyright 2020, Elsevier). For an amorphous carbon, cleaved Kraft‐Lignin was analyzed. For information about the graphite and amorphous carbon reference samples and respective X‐ray scattering and Raman spectroscopy analysis see Supporting Information.

The most prominent allotropic form of carbon as a solid is graphite. The structure of parallelly stacked hexagonal graphene layers consisting of “sp^2^‐hybridized” carbon atoms with a C─C bond length of 1.417 Å is the main part of the definition of graphite by IUPAC.^[^ ^5^ ^]^ Also according to IUPAC, the stacking can be arranged in a rhombohedral or hexagonal way which leads to a 3D crystalline order. The hexagonal stacking is graphically illustrated by the periodically repeated *ABAB* stacking of graphene layers in Figure 1a.^[^ ^5^ ^]^ Due to this 3D long‐range order, characteristic and sharp (*hkl*) reflections arise in the corresponding X‐ray diffraction (XRD) pattern (Figure 2, left). The Raman spectrum is dominated by the G band at ∼ 1570 cm^−1^. It originates from the one‐phonon Raman process with the lattice mode of E_2g_ symmetry at the center of the Brillouin zone (wave vector *q*  =  0). The D band at ∼ 1350 cm^−1^ is due to a double‐resonant Raman process involving a phonon at the K‐point and a defect, *e.g*., a graphene edge defect or a point defect. Typically, its intensity is low in high‐quality graphite.^[^ ^11^ ^]^ Another double‐resonant Raman process involving two lattice phonons of *q* ≠ 0 yields the so called G’ or 2D Raman signal between 2600 and 2800 cm^−1^, which exhibits two bands and is very sensitive to in‐plane crystallite size^[^ ^11^, ^12^, ^13^ ^]^ and out‐of‐plane stacking order^[^ ^14^, ^15^, ^16^, ^17^, ^18^, ^19^, ^20^ ^]^ of graphene layers. The sensitivity is underlined by comparing the Raman spectrum of graphite with the one of graphene consisting of only single layers of carbon atoms in the planar condensed ring system, which presents the basic structural unit for all sp^2^‐type carbon‐based materials.^[^ ^5^ ^]^ The 2D band in the Raman spectrum of graphene consists of a single peak of much higher intensity compared to the spectrum of graphite (Figure S2). With increasing number of stacked graphene layers, the 2D band shifts in position and its shape changes developing a band with four peak components toward evolving the two‐peak characteristic of the 2D band in the Raman spectrum of graphite.^[^ ^21^ ^]^ As carbonaceous materials differ in their stacking order according to IUPAC, Raman spectra can help in identifying them, as this example demonstrated.

Hence, all structural characteristics which lead the IUPAC definition^[^ ^5^ ^]^ of graphite exhibit unambiguous features in the respective XRD pattern and Raman spectrum.

### 2.2 Amorphous Carbon

In general, amorphous carbon is defined by the absence of 2D or 3D long‐range order (Figure 1),^[^ ^5^ ^]^ therefore its structure is the direct counterpart to the highly ordered (= crystalline) structure of graphite within the class of carbonaceous materials. As schematically visualized in Figure 1d, molecular moieties with sp^2^ as well as sp^3^‐configuration, localized *π*‐electrons, and greater deviations in the C─C bond length and the bond angles are present.^[^ ^5^ ^]^ Cleaved Kraft‐Lignin was chosen as representative of amorphous carbons here, as it is a phenolic polymer consisting of various methoxylated phenylpropanoid building blocks.^[^ ^22^ ^]^ The X‐ray scattering pattern (Figure 2, right) does not exhibit any distinct signal of a 2D hexagonal array of carbon atoms, reflecting the absence of three‐ or 2D long‐range order. Applying the Bragg equation, the signal at 2*θ* ≈ 20° corresponds to an average interlayer distance of 0.44 nm, which substantially exceeds typical values for the interlayer distance in graphitic and non‐graphitic carbons, and thus should not be interpreted in terms of graphene stacks. Similarly, only a very broad signal appears in the measured Raman spectrum (see also Figure 2 and Figure S3). In absence of long‐range order, all vibrational modes are localized, and crystal wave vector *q* is not a good quantum number. Various sp^3^ and sp^2^‐bonded species contribute to the observed Raman spectrum causing its broadness with a double‐peak structure between 1000 and 1700 cm^−1^, which essentially reflects the density of states of the localized vibrations.^[^ ^23^, ^24^ ^]^ Details of the Raman spectra of amorphous carbons may vary because of the inherent disorder and irregularity of the structure,^[^ ^25^ ^]^ *e.g*., allowing for different ratios of sp^2^ and sp^3^ configurations or variations of bond lengths and strain. Thus, a deeper analysis of Raman spectra yields additional information about amorphous carbon which cannot be obtained by X‐ray scattering.

### 2.3 Graphitic and Non‐Graphitic Carbon

Being aware of the structure of graphite and amorphous carbon, which clearly set the limits toward a crystalline structure and a disordered polymeric network, helps to understand “graphitic” and “non‐graphitic” carbons and their corresponding “microstructure”, *i.e*., the arrangement and dimension of graphene sheets and their stacking. The lack of crystallographic order in all three dimensions, but a 2D long‐range order within the hexagonal layers defines the class of “non‐graphitic carbons” according to IUPAC,^[^ ^5^ ^],^ *i.e*., general (*hkl*) reflections are absent in the corresponding powder X‐ray or neutron scattering pattern. The X‐ray scattering pattern of non‐graphitic carbons thus exhibits diffuse and overlapping (00*l*) and (*hk*) reflections (Figure 2).^[^ ^28^, ^29^ ^]^ Raman spectra typically exhibit a single peak structure of the 2D signal (Figure 2), reflecting disorder in the stacking of the graphene sheets, *e.g*., in case of “turbostratic” carbon, compared to the two peak 2D signal in graphite.^[^ ^30^, ^31^, ^32^ ^]^ Beyond this formal definition, this peculiar structure is outlined in the following.

**Figure 3:**
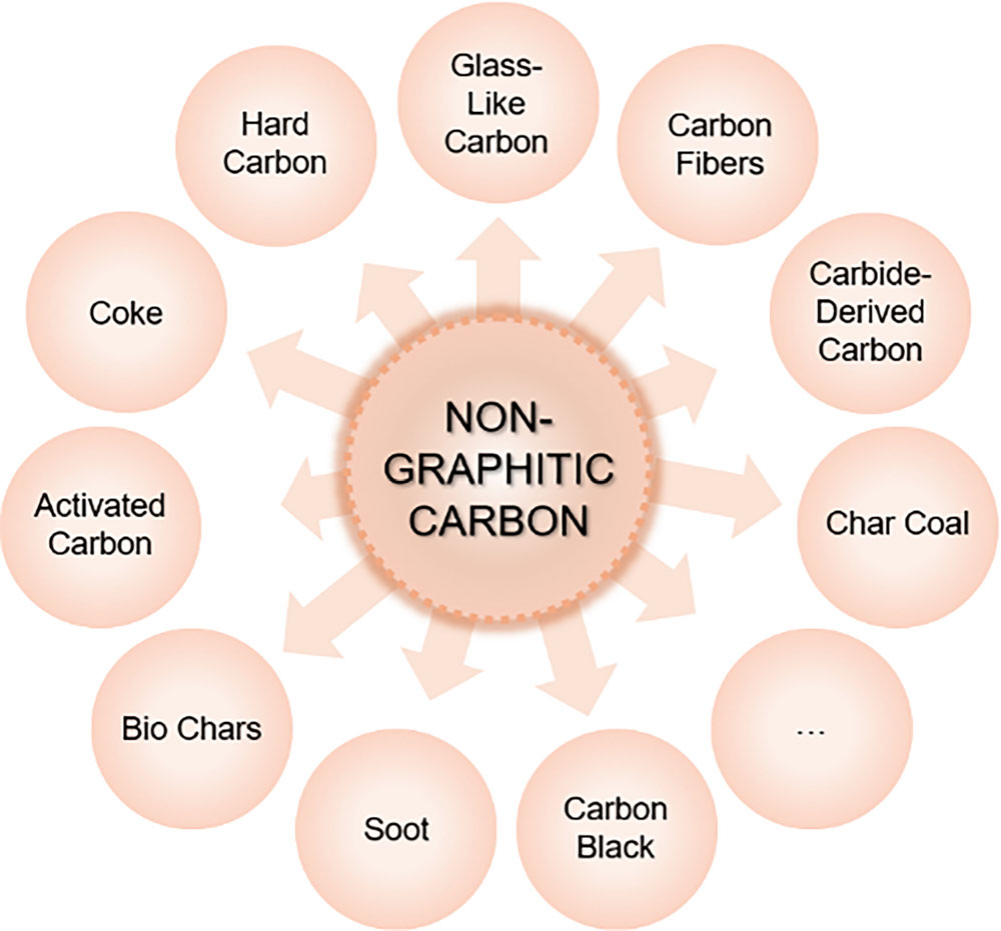
Overview of different commonly used carbon materials which all can be categorized as non‐graphitic carbon. By this, the versatility, variability and significance of non‐graphitic carbon is visualized.

Especially non‐graphitic carbon materials are a rising topic in literature as they are applied in various energy technologies. Several carbon materials with various names and physical‐chemical properties can be classified as non‐graphitic carbon as Figure 3 demonstrates. To name one, glass‐like carbon is a prominent representative with high industrial relevance as it is applied as material for electrodes or crucibles for high‐purity material processing due to properties like chemical inertness or high‐temperature stability.^[^ ^33^, ^34^ ^]^ Similar to the term “hard carbon”, glass‐like carbon received its name due to the macroscopic behavior but the microstructure does not correspond to a glass‐like amorphous state, it is non‐graphitic with graphene layers as basic structural units.^[^ ^10^ ^]^

The microstructure of all these non‐graphitic carbons is of crucial importance for the desired application, but is often misstated as “amorphous” or “graphitic”, while still all of them belong to the category of “non‐graphitic” carbon materials.^[^ ^7^, ^8^ ^]^ However, the microscopic structure of non‐graphitic carbons in terms of the arrangement of graphene‐like layers was already studied and clarified in the middle of the past century. The fact that such carbon materials are not amorphous, but consist of 2D hexagonal carbon layers was shown already by Warren in 1934.^[^ ^35^ ^]^ In subsequent publications, it was proven that graphene layers exhibit 2D long‐range order and are stacked, but can exhibit disorder in the stacking (“translational” disorder) as well as random orientation parallel to the layer. The term of “turbostratic” disorder was coined for this kind of stacking.^[^ ^28^, ^36^ ^]^ Within an individual graphene layer, defects in the planar hexagonal layer, *i.e*., sp^3^‐hybridized carbon atoms, hydroxy groups, or differing C─C bond lengths can occur. This existence of functional groups and foreign atoms as defects can not only affect the macroscopic properties but also strongly influences the stacking height and layer extend as well as their respective disorder as our previous studies showed.^[^ ^37^ ^]^ Figure 1c illustrates the microscopic structure with varying form, orientation, and lateral extension of the graphene‐like layers in non‐graphitic carbons.

**Figure 4:**
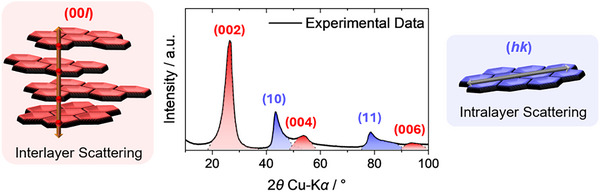
An exemplary wide‐angle X‐ray scattering (WAXS) pattern for non‐graphitic carbons illustrating the two coherent scattering contributions with interlayer scattering causing (00*l*) reflections and intralayer scattering being responsible for (*hk*) reflections. For simplicity, incoherent background contributions were not considered here.

In case of non‐graphitic carbons, the term “diffractogram” should be avoided in X‐ray scattering analysis and, *e.g*., “scattering pattern” should be used instead (Wide‐Angle X‐Ray/Neutron Scattering, “WANS/WAXS”), because of the typically significant structural disorder and concomitant broad reflections. As further distinctive feature, the (*hk*) reflections exhibit an asymmetric shape for powder materials (Figure 2, middle right).^[^ ^38^ ^]^ These (*hk*) and (00*l*) reflections can be assigned clearly to different types of scattering interferences. Intralayer scattering arises from interference occurring within the graphene layers themselves and creates asymmetric (*hk*) reflections, whereas symmetric (00*l*) reflections originate from interlayer scattering where interference occurs between radiation scattered at different graphene sheets of a stack (Figure 4).^[^ ^29^ ^]^ Accordingly, the total scattering intensity is given by a superposition of these two coherent scattering contributions (interlayer and intralayer scattering), and additionally by small‐angle scattering and incoherent Compton scattering as non‐constant background.^[^ ^29^ ^]^

The term “graphitic carbon” should be used as soon as (*hkl*) reflections, which are expected for graphite, start to occur in the diffraction pattern, *i.e*., a 3D graphite structure is partially developed, and crystalline order in the third direction arises.^[^ ^5^, ^39^ ^]^ However, “graphitic” carbons differ from “graphite” in that graphitic carbons do not exhibit the perfect 3D long range order and thus do not present all of the (*hkl*) signals of graphite. According to IUPAC, graphitic carbon is present in the allotropic form of graphite regardless of the homogeneity of the crystalline domains or the existence of structural defects.^[^ ^5^ ^]^ The example of graphitic carbon demonstrates that an extensive characterization is necessary to correctly classify the material. As visible in Figure 2, the Raman spectra of graphite and graphitic carbon are very similar, apart from small differences in the linewidths of the signals. In both samples, the G band dominates the spectra causing an *I*_D_/*I*_G_ intensity ratio near zero. The G’ or 2D Raman signal is asymmetric in both cases which reflects the stacking order.^[^ ^11^ ^]^ The X‐ray scattering patterns show more pronounced differences (Figure 2, top row), thus scattering techniques are better suited for distinguishing between these two types of carbon materials than Raman spectroscopy.

### 2.4 Graphitizable (“*Soft*”) and Non‐Graphitizable (“*Hard*”) Carbon

**Figure 5:**
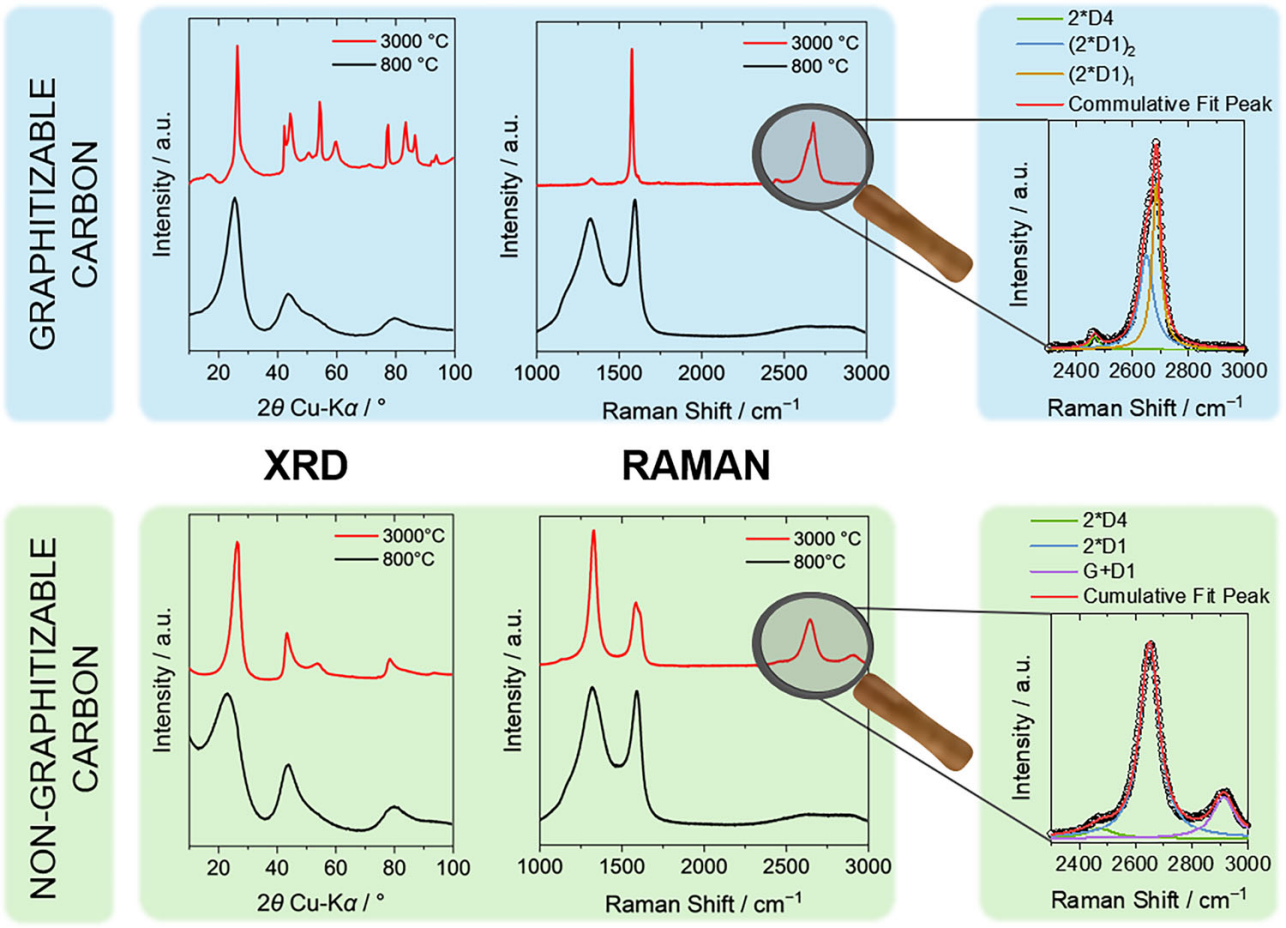
The comparison of X‐ray scattering patterns and Raman spectra of graphitizable (top row) and non‐graphitizable (bottom row) carbons treated at 800 °C (black) and 3000 °C (red). As representative for a graphitizable carbon, a low softening‐point pitch was chosen and the scattering pattern and Raman spectrum were reprinted with permission from Ref. [26]. (Copyright 2016, Elsevier) and with permission under a Creative Commons CC‐BY 4.0 from Ref. [11]. (Copyright 2020, Elsevier). A phenol‐formaldehyde resin‐based carbon serves as example for a non‐graphitizable carbon. The scattering pattern as well as Raman spectrum were reprinted with permission from Ref. [27]. (Copyright 2019, Elsevier) and with permission under a Creative Commons CC‐BY 4.0 from Ref. [11]. (Copyright 2020, Elsevier).

Following the pioneering work of Warren and Biscoe^[^ ^36^ ^]^ Rosalind E. Franklin significantly shaped the field of structural analysis. Mostly known due to her work on the deoxyribonucleic acid (DNA) structure elucidation by demonstrating the double helix form being consistent with the X‐ray diffraction patterns,^[^ ^40^, ^41^ ^]^ she furthermore investigated the structure of different carbonaceous materials.^[^ ^39^ ^]^ She already demonstrated that the order of graphene layers and structural defects within the non‐graphitic carbon material (Figure 1c) are highly dependent on the applied carbonization temperature and carbon precursor. Rosalind E. Franklin established the subdivision of non‐graphitic carbons into “graphitizable/graphitizing” and “non‐graphitizable” carbons. The latter are nowadays most commonly known as “hard carbon” as synonymous expression although she did not use the term “hard carbon” herself.^[^ ^39^ ^]^ The structural differences between “graphitizable/graphitizing” and “non‐graphitizable” carbons are demonstrated by the scattering patterns and Raman spectra in Figure 5. For a low softening point pitch (Figure 5, blue top row) treated at 800 °C (black), the experimental data clearly indicate a non‐graphitic character by broad and overlapping (*hk*) and (00*l*) reflections in the X‐ray scattering pattern, and a high Raman signal intensity of the defect‐activated D band at ∼ 1350 cm^−1^. Also, the second‐order 2D band is only weakly developed, confirming the onset of stacking of the disordered graphene‐like layers. Heat treatment of the same precursor at 3000 °C (red) yields a material exhibiting (*hkl*) reflections typical of a graphitic carbon, *i.e*., the asymmetric (10) reflection splits up into separate and symmetric reflections. In the Raman spectrum, the D band intensity strongly decreases. The 2D signal gets more pronounced and exhibits an asymmetric line shape due to the splitting into two peaks, as highlighted in Figure 5, reflecting the graphene layer ordering into sp^2^‐hybridized graphene stacks.^[^ ^11^ ^]^ Hence, non‐graphitic carbon was converted into graphitic carbon by high‐temperature treatment, which defines this type of non‐graphitic carbon as graphitizable carbon.^[^ ^5^ ^]^ Heat treatment at even higher temperatures would finally convert this graphitic carbon into graphite. Based on IUPAC definitions, graphite produced by graphitizing of non‐graphitic carbons is called “synthetic” graphite.^[^ ^5^ ^]^ The main differences compared to natural graphite presents the lower impurity content making it attractive for technologically important high‐purity demanding applications like steelmaking electrode bars or crucibles.^[^ ^42^ ^]^ Non‐graphitic, graphitizable carbons are also called “soft” carbons as synonym.^[^ ^43^ ^]^

In contrast, even applying a temperature of 3000 °C at atmospheric or lower pressure, a non‐graphitic resol‐based carbon (Figure 5, green bottom row) cannot be converted into graphitic carbon without additional catalyst and is therefore classified as non‐graphitizable carbon.^[^ ^5^ ^]^ The (*hk*) and (00*l*) reflections in the scattering pattern become sharper, less overlapping and more symmetric for a material treated at 3000 °C (red) compared with a material carbonized at 800 °C (black), but no (*hkl*) reflections are observed, thus, no graphitic structure arises. Treatment at a higher temperature leads to in‐plane growth of existing layers incorporating non‐organized carbon atoms causing enlarged polyaromatic domains and reduced number of defects.^[^ ^27^, ^37^ ^]^ Combining elemental analysis with microstructural evaluation of high‐quality WANS data, applying the later mentioned fitting approach, showed that a lower amount of foreign atoms as defects is correlated with an increased stack/layer size and order.^[^ ^37^ ^]^ Accordingly, though to a lower extent, also an increase in the stacking height and the stacking order of graphene stacks takes place, as for all non‐graphitic carbons.^[^ ^27^ ^]^ These microstructural processes do not lead to a perfect graphite structure but only shape the scattering patterns toward more defined (*hk*) and (00*l*) reflections. The non‐graphitizing character can also be seen in the Raman spectrum as the 2D band consists of a single peak line shape even after a heat treatment at 3000 °C.^[^ ^11^ ^]^ In energy materials applications, the term of “hard carbon” is now established for this type of non‐graphitizable materials within the class of non‐graphitic carbons.

**Figure 6:**
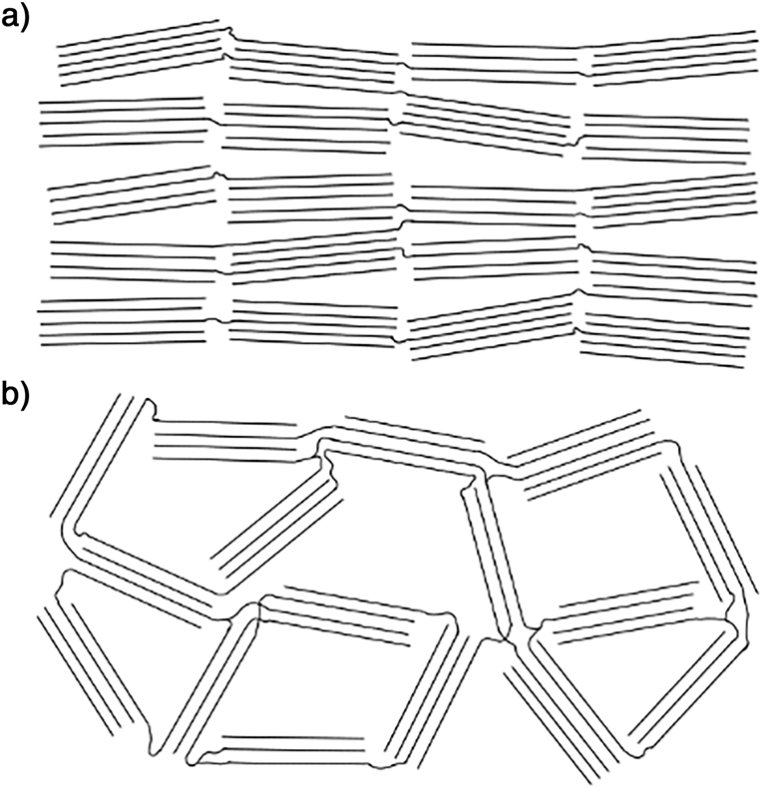
Schematic illustration of microstructural arrangement within a) graphitizable and b) non‐graphitizable carbons in accordance with the structure model by Rosalind E. Franklin.^[^ ^39^ ^]^

Whether a carbon material is graphitizable or not, thus mainly depends on the applied carbon precursor. Coal tar pitches or petroleum‐based pitches are easily graphitized while resins originating from hydroxybenzenes (*i.e*., resorcinol, phloroglucinol, or bioprecursors like lignin) being thermopolymerized with an aldehyde as linking‐agent represent non‐graphitizing carbon precursors. Again, Rosalind E. Franklin proposed the first structure model stating that non‐graphitizing carbon precursors hinder a vast rearrangement of the graphene‐like layers due to a high degree of cross‐linking (Figure 6b). By contrast, weakly cross‐linked graphitizing precursors tend to develop nearly parallel graphene stacks (Figure 6a) with less steric demand during rearrangement toward a graphitic structure with increased stacking height and graphene layer extent, transforming into graphitic carbon and finally graphite.^[^ ^27^, ^39^ ^]^

Rosalind E. Franklin's structural proposal also pictures key characteristics of non‐graphitic and non‐graphitizable carbons which are a mass density being significantly below that of graphite of 2.26 g cm^−3^ and an inaccessible intrinsic microporosity.^[^ ^39^, ^43^ ^]^ The illustrated model of non‐graphitic carbons might be outdated as the cross‐linking structures must be uncommonly rigid and strong to hinder a conversion into extended graphene stacks at temperatures above 3000 °C, but it is still useful to visualize and underline the differences between the different non‐graphitic carbon materials also in contrast to an amorphous carbon. The complexity of the structure elucidation of non‐graphitizing carbons is stressed by the various different structural models that scientists presented since Rosalind Franklin's first attempt. Recent models for non‐graphitizable carbons assume a ribbon‐like structure or fragments of randomly curved carbon sheets as building blocks.^[^ ^27^, ^43^, ^44^, ^45^ ^]^ Hence, refining the structural models is still a matter of active research.

## 3 State‐of‐the‐Art Structural Characterization of Non‐Graphitic Carbons

A distinct understanding of the atomic structure of non‐graphitic carbon materials is indispensable for describing and characterizing them correctly as the previously explained turbostratic structure directly influences the recorded powder scattering patterns (Figure 2, middle right) causing broad, asymmetric and overlapping (*hk*) and (00*l*) reflections. Probably as a consequence of the appearance of such an uncommon and apparently “lousy” scattering patterns, three misconceptions about “hard carbon” entered literature of carbon research and are listed below:

- “Hard carbon” is associated with an amorphous microstructure.^[^ ^7^, ^8^ ^]^
- The graphene layer arrangement in “hard carbon” is described as highly disordered.^[^ ^46^, ^47^ ^]^
- “Hard carbon” is labelled as graphitic carbon.^[^ ^9^, ^48^ ^]^

**Figure 7:**
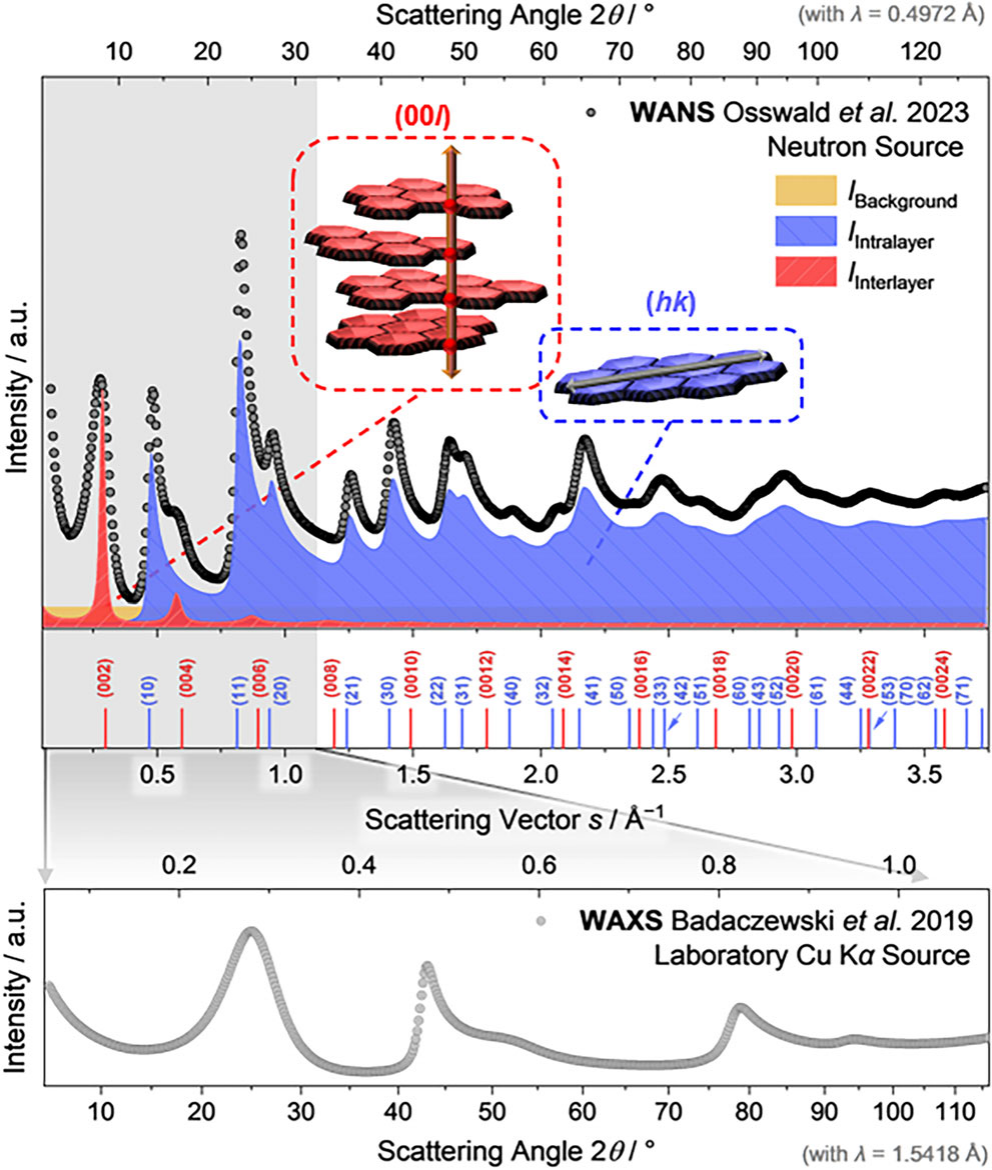
Experimental WANS data measured at a wavelength of *λ*  =  0.4972 Å of a phenol‐formaldehyde resin (top) treated at 2100 °C as non‐graphitic carbon representative reprinted with permission under a Creative Commons CC‐BY 4.0 from Osswald *et al*.^[^ ^37^ ^]^ (Copyright 2023, MPDI) with additional breakdown of interlayer (red), intralayer (blue), and background (grey) scattering contributions. In comparison, the WAXS pattern measured at a wavelength of *λ*  =  1.5418 Å is reprinted with permission from Badaczewski *et al*.^[^ ^27^ ^]^ (Copyright 2019, Elsevier) of the same sample (bottom) in order to highlight the significantly smaller number of reflections which can be recorded with a laboratory Cu K*α* setup.

These misconceptions shall be cleared up to avoid a potential chain of errors in literature. Incorrect terminology in literature makes it difficult for authors to describe their carbon material correctly. Misconception *I* can be resolved easily by comparing the X‐ray scattering patterns of a non‐graphitic carbon (Figure 2, middle right) with the pattern of an amorphous carbon (Figure 2, right). The non‐graphitic carbon reflections are broad but can be assigned to characteristic (*hk*) and (00*l*) reflections whereas amorphous carbon shows no distinct reflections of a 2D hexagonal array of carbon atoms at all. Second, misconception *II* can be clarified by using standard laboratory X‐ray diffraction instruments and also using neutron scattering. For X‐ray scattering, the atomic form factor results in substantial damping of the scattering curve with increasing scattering vectors, while the atomic form factor is almost constant for neutron scattering. Figure 7 shows that in wide‐angle neutron scattering (WANS) patterns the systematic and characteristic (*hk*) intralayer reflections are thus visible up to scattering vectors exceeding *s* > 3 Å^−1^ due to the high order and regularity of the graphene layers.^[^ ^37^ ^]^ Lastly, looking at the scattering patterns is sufficient again to refute the assumption of a graphitic structure of “hard carbon” (misconception *III*). For graphitic carbon, the scattering pattern (Figure 2, middle left) must exhibit (*hkl*) reflections corresponding to the 3D long‐range order. For “hard carbons”, which are represented by the scattering pattern of a non‐graphitic carbon, there is no 3D long‐range order, which results in the absence of general (*hkl*) reflections (Figure 2, middle right).

After the discussion of these misconceptions and pointing out the structural properties of non‐graphitic carbons such as “hard carbon”, suitable and recommendable state‐of‐the‐art analysis of these materials shall be addressed. The materials characteristics leading to broad and overlapping reflections make conventional Scherrer‐type analysis of diffraction patterns inadequate for an elaborate and reliable analysis of the carbon microstructure, as this frequently used approach relates the widths of signals solely to the domain size which is obsolete for non‐graphitic carbon. Also, the determination of the average interlayer spacing ${\bar{a}}_{3}$ based on the position of the (00*l*) reflections with Bragg´s law is systematically erroneous as the positions are affected by several effects, *e.g*., the superposition with neighboring (*hk*) reflections.^[^ ^29^ ^]^ Also, line‐broadening is caused by size and disorder effects, the latter often are not being properly taken into account.^[^ ^27^, ^49^ ^]^ State‐of‐the‐art structural evaluation by WAXS/WANS is based on fitting theoretical model functions to the experimental scattering data. Several approaches all relying on the same principle of calculating a scattering profile by varying different model parameters describing the carbon microstructure are in use. The algorithms by Dopita *et al.*,^[^ ^50^ ^]^ Fujimoto and Shiraishi,^[^ ^51^ ^]^ Shi and Dahn,^[^ ^52^ ^]^ or Ruland and Smarsly^[^ ^29^ ^]^ should be named here as prominent examples. These approaches assume the observed scattering curve as superposition of incoherent background scattering and coherent scattering which is itself composed of interlayer scattering and intralayer scattering contributions, as illustrated in Figure 4.^[^ ^29^ ^]^ By adjusting microstructural parameters, the continuous model function is fitted to the experimental WAXS/WANS data minimizing the difference between the theoretical scattering function and the experimental pattern. As a result, quantitative parameters describing the layers’ structure, *i.e*., the graphene layer extent *L*_a_ or the average C─C bond length *l*_cc_, and stacking structure, *i.e*., the average stacking height *L*_c_ and the average interlayer spacing ${\bar{a}}_{3}$, are obtained.^[^ ^29^, ^38^, ^53^ ^]^ With the implementation of the algorithm of Ruland and Smarsly^[^ ^29^ ^]^ in calculation programs like *Wolfram Mathematica*,^[^ ^38^ ^]^ *CarbX*,^[^ ^54^ ^]^ or *GNU Octave*,^[^ ^53^ ^]^ the structure evaluation based on this approach is applicable, even as open‐access software. Only a reliable microstructural data evaluation results in meaningful structural information and enables further investigations such as, for instance, correlating the microstructure with observed macroscopic material properties in various applications or studying the influence of elemental composition on structural rearrangement as shown in Figure S4 by Osswald *et al*.^[^ ^37^ ^]^ Hence, the usage of such fitting approaches instead of the Scherrer‐type analysis is highly recommended for non‐graphitic carbon characterization based on scattering analysis.

Regarding structure evaluation by Raman spectroscopy, empirical models for analyzing the average graphene layer extent *L_a_* (or crystallite size) of non‐graphitic and graphitic carbons have been established and steadily improved.^[^ ^12^, ^13^, ^23^, ^55^ ^]^ The most common analysis is based on the intensity ratio of the defect‐activated D and G Raman modes. Here, the Tuinstra and Koenig relation of *I*_D_/*I*_G_ ∝ *L*_a_^−1^ is valid for crystallites sizes with *L*_a_ >> 2 nm, whereas the Ferrari and Robertson relation with *I*_D_/*I*_G_ ∝ *L*_a_^2^ is within the scope of validity if *L*_a_ falls below the transition region at ∼ 2 nm.^[^ ^12^, ^23^ ^]^ Analyzing non‐graphitic carbon, combining the two relations yields a master curve^[^ ^56^ ^]^ *I_D_*/*I_G_* versus *L_a_* over a large range of *L_a_* values, which then enables determining *L*_a_ from given *I_D_*/*I_G_* values. However, Schuepfer *et al*.^[^ ^11^ ^]^ pointed out that this master curve is not universally applicable. First, this is due to the two aforementioned effects causing the *I_D_*/*I_G_* versus *L*_a_ dependance necessarily exhibiting a maximum, *i.e*., the determination of *L*_a_ is not unambiguous. Second, the D band intensity depends not only on the defect density, but also on the type of defect and the Raman excitation wavelength and, thus, on the precursor and carbonization procedure used as well as experimental conditions, *i.e*., *I*_D_/*I*_G_ cannot be considered an intrinsic property as it is defect‐related.^[^ ^11^ ^]^ Nevertheless, the approach is useful and fast when comparing samples with the same defect structure provided the *L_a_* scale is verified by WAXS or WANS.^[^ ^11^, ^37^, ^57^ ^]^ An alternative, essentially intrinsic approach is based on the dependence of the G and D band position as well as their line shape on the lateral size *L*_a_. The band positions are independent of defects and, hence, independent of the applied precursor. Thus, only the electronic and vibrational band structure determines the frequency positions allowing precise calculations and correlations with *L*_a_.^[^ ^11^ ^]^ Corresponding phonon confinement models^[^ ^58^ ^]^ which yield the Raman line shape as a function of crystallite size *L_a_* can be used to obtain master curves for the line shape of G (and D mode in case of all graphene‐layer based carbons with *L_a_* > 2 nm). They require a parameterization of the phonon dispersion of graphite, *e.g*., derived by DFT calculations, and a verification of the *L_a_* scaling as the envelope function of the confined phonon model needs to be validated. This scaling is achieved by correlating *L*_a_ values quantified by the WAXS fitting with the G Raman band position.^[^ ^11^, ^37^ ^]^ Such theoretical master curves for the G band position versus *L*_a_ (see Figure S5) have been successfully employed for non‐graphitic and graphitic carbons.^[^ ^11^, ^37^ ^]^ Also the D band position can be correlated with *L_a_*. Here, the laser excitation wavelength dependence of the D band position can be used to validate calculated phonon dispersions and correlations with *L_a_* by determining the D band position at different applied wavelengths.^[^ ^11^ ^]^ Therefore, state‐of‐the‐art characterization of non‐graphitic carbons based on Raman spectroscopy goes beyond the sole consideration of the intensity ratio of the D and G Raman modes and should also include frequency position based correlations to gain quantitative information about the lateral size *L_a_*.

## 4 Practical Guideline on Structural Characterization of Carbon‐Based Materials

**Figure 8:**
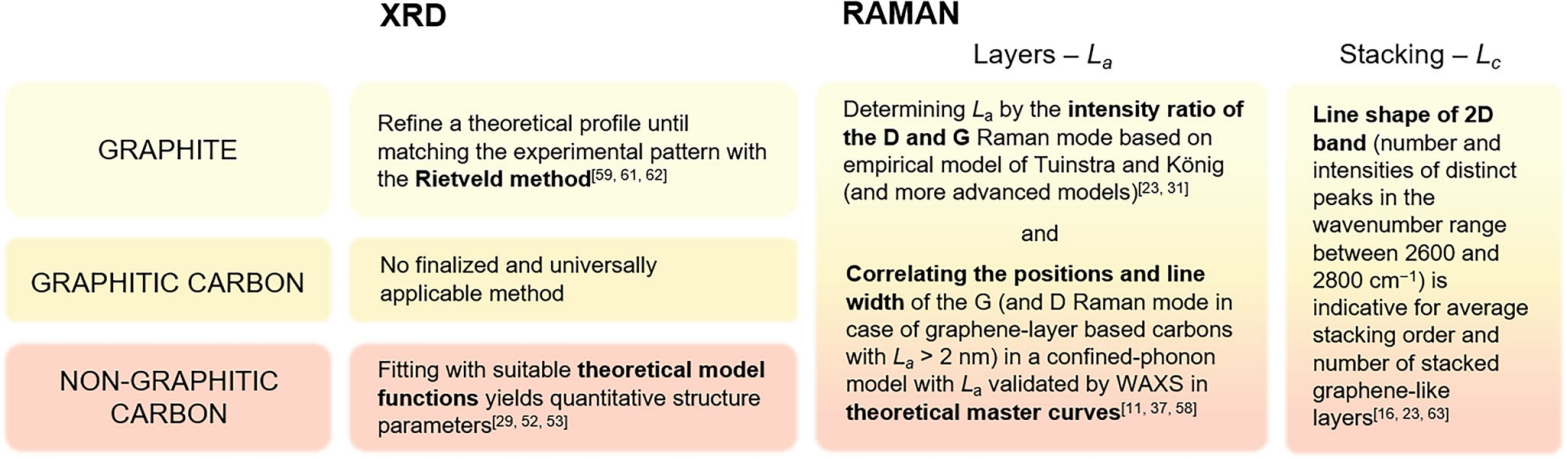
Overview of the practical evaluation of XRD and Raman data of graphite, graphitic carbon, or non‐graphitic carbon materials toward a valid structure elucidation.

Figure 8 provides an overview of the discussed carbonaceous materials with the corresponding and valid characterization approach in order to give guidance for the structural analysis of these materials. References to original literature provide access to further information on the respective techniques.

One option for a state‐of‐the‐art X‐ray or neutron‐based analysis of graphite is Rietveld refinement of the respective diffraction data. In principle, a theoretical diffraction pattern is calculated based on its crystal structure and fitted to the experimental data under variation of structural parameters such as the positions of the atoms, *resp*., ions in the unit cells, crystallite dimension, etc.^[^ ^59^, ^60^, ^61^, ^62^ ^]^ Similarly, Raman spectra of graphite are analyzed by assigning the Raman active modes based on a group theoretical analysis of the crystal structure. The peculiar electronic structure of graphene yields additional features in the Raman spectra such as D and 2D modes due to double‐resonant Raman effects, which require caution in the structural analysis.^[^ ^63^ ^]^ For correlations with the lateral size *L_a_*, the Tuinstra and Koenig relation of *I*_D_/*I*_G_ ∝ *L*_a_^−1^ can be applied since the crystallite size in graphite is typically quite large with *L*_a_ >> 2 nm, as described previously.^[^ ^12^, ^23^ ^]^

For non‐graphitic carbons, the recommended way of evaluating a WAXS pattern is already described in chapter 3. Although also explained in the previous chapter in detail, their structure evaluation based on Raman spectroscopy can be narrowed down to four parameters: The intensity ratio of the defect‐activated D and G Raman modes *I*_D_/I_G_ and its relation to *L_a_* is meaningful for samples with the same defect structure synthesized from the same precursor.^[^ ^11^ ^]^ However, due to their non‐dependence on defects and their direct correlation to the electronic and vibrational band structure, the position of the D and G band, their linewidth and wavelength dependence–in case of the D band–are more universal parameters for determining *L*_a_. Corresponding theoretical master curves for the G band position versus *L*_a_ were determined by Osswald *et al*.,^[^ ^37^ ^]^ for instance. In addition to non‐graphitic carbons, these approaches are also reasonable for the determination of the graphene extension *L*_a_ of graphitic carbons, which represents an advantage of the structure analysis based on Raman spectroscopy. Considering the structure evaluation of graphitic carbons based on X‐ray scattering analysis, neither of both discussed methods (Rietveld refinement or theoretical WAXS data fitting) is applicable for this type of material, since graphitic carbon is structurally located between the two cases of graphite and non‐graphitic carbon. Here, it is recommended to analyze the widths of (00*l*) and (*hkl*) reflections separately to determine *L*_c_, *L*_a_, etc.

Our elaboration makes clear that Raman spectra and scattering patterns of carbonaceous materials yield complementary information and exhibit different strengths and weaknesses. Neither for the former nor the latter, a universal model capable of comprehensively describing the structural changes from crystalline graphite to amorphous carbon with intermediate graphitic or non‐graphitic materials exists. The quest for such a universally applicable model is still on. Combining the strengths of Raman spectroscopy and scattering methods to assess structural properties of carbonaceous materials and relating them in structural models may be a viable way forward. Going beyond the here mentioned methods, also HRTEM is a promising technique to analyze the discussed materials. It presents certain drawbacks, for instance, as it is not available as routine technique. Also, it represents a local method not representing the bulk material, and a high energy electron beam may induce microstructural changes of the analyzed sample.^[^ ^10^, ^64^ ^]^ Additionally, limited resolution accuracy or overlapping structures can limit the data acquisition and extraction. Nevertheless, also by the increasing automatization and computer algorithm‐based lattice fringe analysis, HRTEM can provide complementary structure quantification and will become more relevant in future.^[^ ^65^ ^]^ Thus, there is still need for research dealing with carbon characterization regardless of the chosen analytical method, although these materials already have been an essential part in materials research and application for a long time.

## 5 Conclusion

In the context of the worldwide striving toward environmentally friendly energy supply with reduced CO_2_ emissions, carbonaceous materials are of increasing relevance. Yet, one should be aware of the structural differences, valid characterization techniques, and correct classifications, all often neglected in current literature. Certainly, it is debatable, if applying the correct IUPAC‐based classification is mandatory. Indeed, if a non‐graphitic carbon, *resp*., “hard carbon” sample is titled as “amorphous”, but the respective researchers have a correct picture of graphene layer assembly in mind, and characterize the material in a proper way, applying a wrong terminology is just a formality and does not endanger the validity of any conclusions on a structure‐property relationship. However, scientific progress might potentially be hindered or even impeded, if wrong structural models are assumed due to incorrect terminology, and false interpretation of experimental results arises. In our view, categorizing a given carbon material with respect to the different types of carbon‐based materials is only meaningful, if the corresponding correct microstructure is connected to it. Also, new categories of carbon materials should not be introduced solely based on macroscopic properties or new synthetic procedures, if it is not accompanied by proper assignment in terms of IUPAC nomenclature.

## Supporting Information

The authors have cited additional references within the Supporting Information.^[^ ^5^, ^11^, ^21^, ^24^, ^25^, ^37^, ^66^ ^]^

## Conflict of Interests

The authors declare no conflicts of interest.

## Supporting information



Supporting Information

## Data Availability

The raw data of this study are openly available with the following link: https://doi.org/10.22029/jlupub‐20438.
