# Supplementary material for: Between Order and Confusion: Clearing up Structural Misconceptions in Carbon Materials Nomenclature
Source: Angew Chem Int Ed Engl. 2026 Feb 3;65(11):e19975. doi: 10.1002/anie.202519975 (PMC12970515; doi:10.1002/anie.202519975)
Supplement: Supplementary file 1 — Supporting Information [file ANIE-65-e19975-s001.docx]

Between Order and Confusion: Clearing Up Structural Misconceptions in Carbon Materials Nomenclature

Chantal Glatthaar^[a]^, Felix Badaczewski^[a,b]^, Peter J. Klar*^[c]^, Bernd M. Smarsly*^[a]^

[a] Prof. Dr. B. M. Smarsly, C. Glatthaar, Dr. F. Badaczewski
Institute of Physical Chemistry
Center of Materials Research
Justus Liebig University Giessen
35392 Giessen, Germany
E-mail: bernd.smarsly@phys.chem.uni-giessen.de

[b] Dr. F. Badaczewski

Schunk Kohlenstofftechnik GmbH
35452 Heuchelheim, Germany

[c] Prof. Dr. P. J. Klar
Institute of Experimental Physics I
Center of Materials Research
Justus Liebig University Giessen
35392 Giessen, Germany
E-mail: peter.j.klar@exp1.physik.uni-giessen.de

**Supporting Information**

**Materials**

As representative sample of Graphite, graphite flakes (99% carbon basis, -325 mesh particle size (50 – 70%), natural) from *Sigma-Aldrich* was used and analyzed by X-ray scattering and Raman spectroscopy. For an amorphous carbon, cleaved Kraft-Lignin extracted from spruce and pine wood was provided by the Fraunhofer Center for Chemical-Biotechnological Processes (CBP) in Leuna, Germany.

**
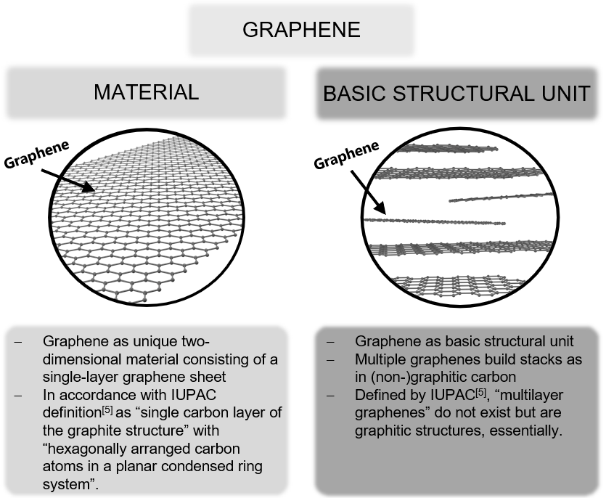
Comparison of Graphene as Material with Graphene as Single Carbon Layer and Basic Structural Unit for sp^2^-Type Carbon Based Materials**

**Figure S1.** Schematic illustration of graphene as two-dimensional material consisting of only one single layer of hexagonally arranged sp^2^-type carbon atoms as defined by IUPAC^[5]^ (left) and as basic structural unit in carbon materials build by stacking of graphene layers.

**
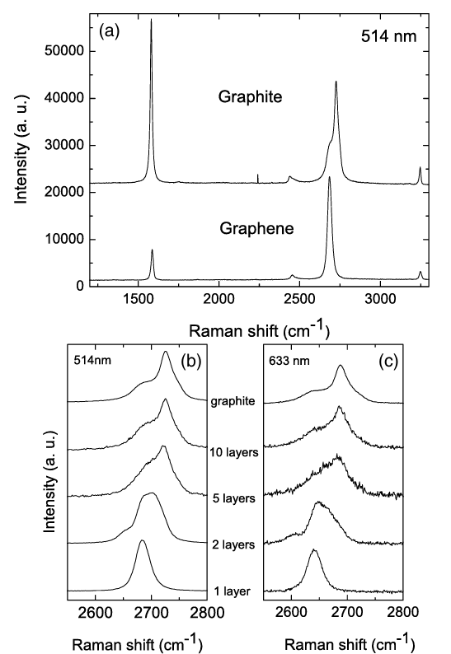
Comparison of Raman Spectra of Graphite and Graphene**

**Figure S2.** a) Raman spectra of graphite (top) and graphene (bottom). The graphics b) and c) show the evolution of the 2D band in the spectra depending on the number of stacked graphene layers for measurements with λ = 514 nm and λ = 633 nm. This demonstrates the sensitivity of the Raman spectroscopy towards the stacking of different carbon-based materials. The Figure was reprinted with permission from ref^[21]^ (Copyright American Physical Society 2006).

**Characterization of Representative Graphite and Amorphous Carbon Samples**

For both samples, wide angle X-ray scattering measurements were carried out with a *XRDynamic 500* diffractometer from *Anton Paar* in Brag-Brentano geometry at room temperature. 50 mA and 40 kV with a Cu-K_α1_ radiation (λ = 1.5406 Å) and Cu-K_α2_ radiation (λ = 1.5444 Å) in a 1:2 ratio were applied. For the graphite sample, operation of the measurement was done within the range of 10° < 2*θ* < 115° with a step size of 0.05°, for the amorphous carbon sample a step size of 0.1° was used.

For Raman spectroscopy, a *Renishaw inVia* Raman microscope system in backscattering geometry was used at room temperature with a 50 x objective. In case of graphite, a laser excitation wavelength of *λ* = 515 nm and laser power of 5 mW was applied. 5 accumulations of 10 s have been the integral exposure time within a spectral range of 100 – 3200 cm^−1^. For the amorphous Kraft-Lignin sample, the laser excitation wavelength was λ = 633 nm with a laser power of 5 mW. The integral exposure time was 4 accumulations of 10 s. The spectral range covered 150 – 4000 cm^−1^. Due to the known auto-fluorescence of lignin upon excitation in the visible range of light,^[3]^ intense fluorescent background contributions overlaid the Raman spectrum. The background was therefore subtracted, as shown in **Figure S3**. By that, a representative Raman spectrum for an amorphous carbon sample was obtained similar to the spectra reported by Dresselhaus *et al*.^[24]^, Agarwal *et al*.^[25]^ or Ferrari and Robertson^[66]^.


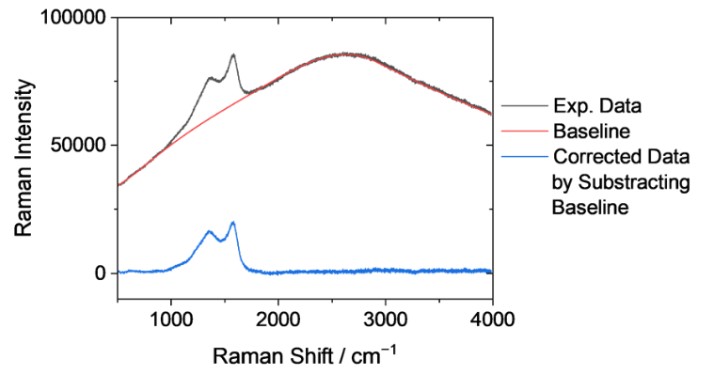


**Figure S3.** Raman spectroscopy data correction for the Kraft-Lignin amorphous carbon sample.

**Correlation Between Elemental Composition and Microstructural Graphene Layer Extension and Disorder**

As Osswald *et al*.^[37]^ showed and is depicted in **Figure S4**, there is a correlation between the elemental composition and the disorder and size of the graphene layers in non-graphitic carbons. The lower the concentration of hydrogen / oxygen / nitrogen impurities, the more extended and ordered are the graphene layers and stacks.^[37]^ Such a correlation is only possible based on a reliable microstructural analysis and data evaluation underlining the importance of a state-of-the-art structural characterization of non-graphitic carbons.

**
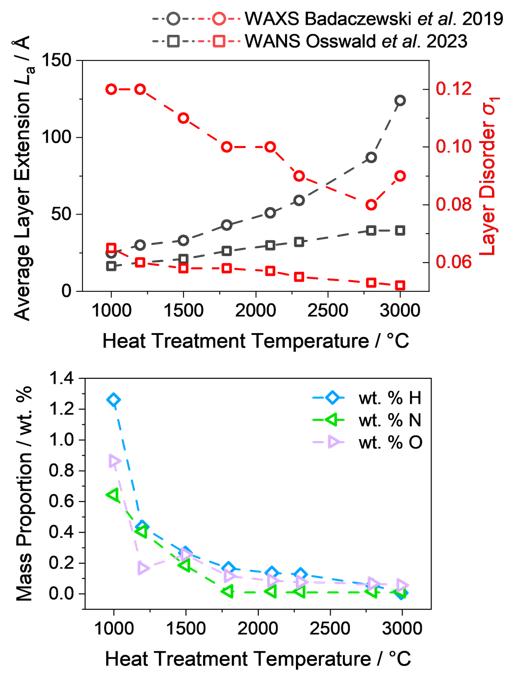
**

**Figure S4.** The average layer extension *L*_a_ and layer disorder *σ*_1_ in a phenol-formaldehyde resin depending on the temperature of the heat treatment is correlated with the mass proportion of the foreign atoms of oxygen, hydrogen and nitrogen. As these atoms are present in form of functional groups in the structure, they hinder the rearrangement of graphene layers towards lateral growth. With less foreign atom concentration, the non-graphitic carbon structure gets more ordered with larger graphene layers. Data is reprinted with permission under a Creative Commons CC-BY 4.0 from ref^[37]^ (Copyright 2023 MDPI).

**Master Curves Correlating L_a_ Values Quantified by WAXS/WANS Fitting With Raman G Band Position**

**
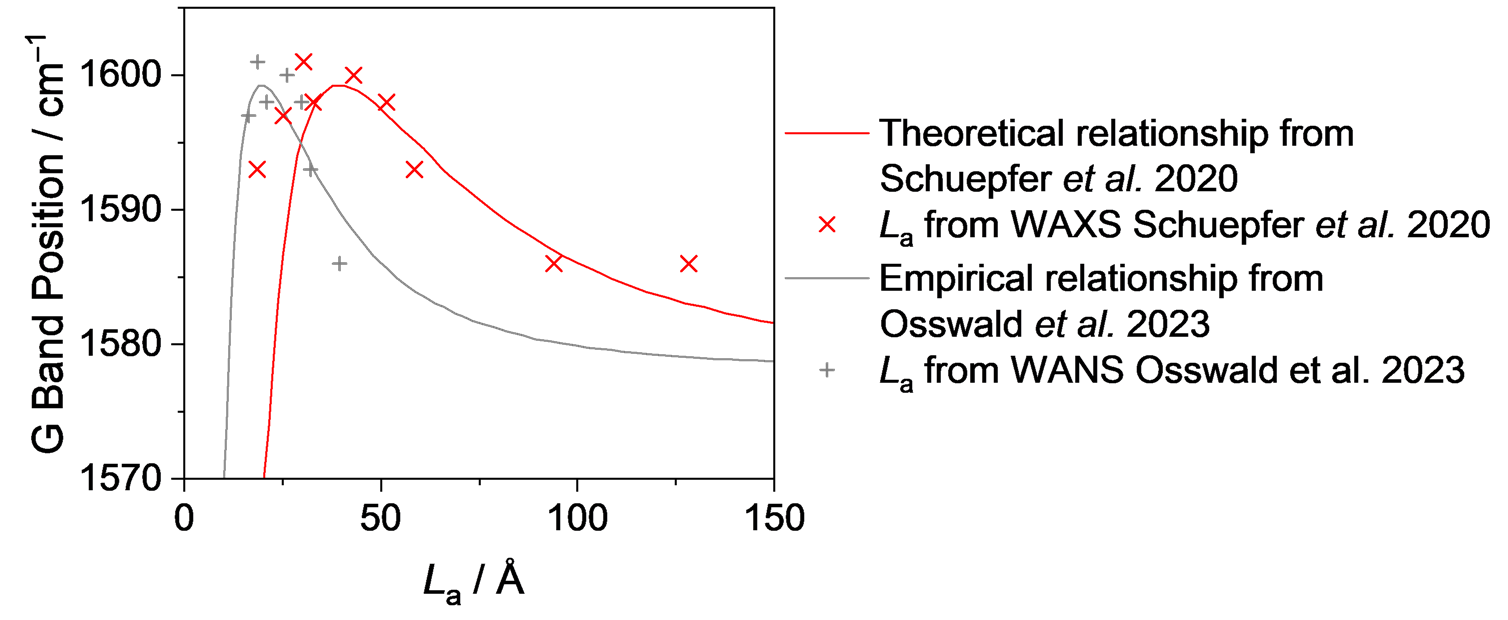
**

**Figure S5.** The Raman G band position is correlated to the graphene layer extension *L*_a_ by the theoretical relationship from Schuepfer *et al*. 2020 with *L*_a_ acquired by WAXS and empirical relationship from Osswald *et al*. 2023 based on WANS evaluation of *L*_a_. Data is reprinted with permission under a Creative Commons CC-BY 4.0 from ref^[37]^ and ref^[11]^ (Copyright 2023 MDPI and 2020 Elsevier).

**References**

[5] E. Fitzer, K.-H. Kochling, H. P. Boehm, H. Marsh, Recommended terminology for the description of carbon as a solid (IUPAC Recommendations 1995), *Pure Appl. Chem.* **1995**, *67*, 473.

[11] D. B. Schuepfer, F. Badaczewski, J. M. Guerra-Castro, D. M. Hofmann, C. Heiliger, B. Smarsly, P. J. Klar, Assessing the structural properties of graphitic and non-graphitic carbons by Raman spectroscopy, *Carbon* **2020**, *161*, 359.

[21] A. C. Ferrari, J. C. Meyer, V. Scardaci, C. Casiraghi, M. Lazzeri, F. Mauri, S. Piscanec, D. Jiang, K. S. Novoselov, S. Roth et al., Raman spectrum of graphene and graphene layers, *Phys. Rev. Lett.* **2006**, *97*, 187401.

[24] M. S. Dresselhaus, A. Jorio, M. Hofmann, G. Dresselhaus, R. Saito, Perspectives on carbon nanotubes and graphene Raman spectroscopy, *Nano Lett.* **2010**, *10*, 751.

[25] U. P. Agarwal, J. D. McSweeny, S. A. Ralph, FT–Raman Investigation of Milled-Wood Lignins: Softwood, Hardwood, and Chemically Modified Black Spruce Lignins, *J. Wood Chem. Technol.* **2011**, *31*, 324.

[37] O. Osswald, M. O. Loeh, F. M. Badaczewski, T. Pfaff, H. E. Fischer, A. Franz, J.-U. Hoffmann, M. Reehuis, P. J. Klar, B. M. Smarsly, On the Highly Ordered Graphene Structure of Non-Graphitic Carbons (NGCs)—A Wide-Angle Neutron Scattering (WANS) Study, *C* **2023**, *9*, 27.

[66] A. C. Ferrari, J. Robertson, Resonant Raman spectroscopy of disordered, amorphous, and diamondlike carbon, *Phys. Rev. B* **2001**, *64*.
